# Two-Dimensional Simulation on the Critical Diameter of Particles in Asymmetric I-Shaped DLD Arrays

**DOI:** 10.3390/mi16030270

**Published:** 2025-02-27

**Authors:** Jiangbo Wu, Zihan Yan, Yongqing He, Jie Liu, Yao Lv

**Affiliations:** 1 School of Energy and Power Engineering, Lanzhou University of Technology, Lanzhou 730050, China; wujb@lut.edu.cn (J.W.); juveforever1896@163.com (Z.Y.); ahriempress1314@gmail.com (J.L.); ly@lut.edu.cn (Y.L.); 2 Chongqing Key Laboratory of Micro-Nano System and Intelligent Sensing, Chongqing Technology and Business University, Chongqing 400067, China

**Keywords:** deterministic lateral displacement, finite element method, critical separation size, micro fluids

## Abstract

Deterministic lateral displacement (DLD) is a passive particle separation method based on microfluidic technology, with its separation mechanism primarily relying on particle size differences. Therefore, the critical separation size is of great significance in the design of DLD devices. The geometric asymmetry of the pillar array design significantly influences fluid behavior and critical particle size variations. This study first analyzed particle motion characteristics through particle trajectory observation experiments within asymmetric microfluidic chips. Subsequently, a two-dimensional numerical simulation method was employed to investigate the effects of three different ratios of lateral gap size to downstream gap size (G_x_:G_y_) on particle trajectories and flow field distribution. The results indicate that as G_x_:G_y_ decreases, the upward flow rate gradually reduces, accompanied by changes in the flow field velocity distribution, causing particles to favor displacement mode. This study provides new theoretical foundations for the precise regulation of particle motion behavior and introduces novel insights for optimizing DLD device design.

## 1. Introduction

Research on the separation of micron-sized particles is of great significance in fields such as materials engineering, medical diagnostics, environmental monitoring, and drug development [1,2,3,4]. Traditional particle separation methods, such as fluorescence labeling separation [5,6,7] and density gradient centrifugation [8,9], face issues such as low separation efficiency, high instrument costs, sample contamination, and loss [8,9,10]. Microfluidics is an emerging technology that precisely controls fluids or samples at the micron level. With its advantages of simple design, rapid analysis, and high sensitivity [11,12,13], microfluidics has become a focal point in particle sorting research in recent years.

Microfluidic particle sorting methods mainly include inertial force sorting [14], surface acoustic wave sorting [15], dielectrophoretic sorting [16], magnetic sorting [17], optical sorting [18], and deterministic lateral displacement (DLD) sorting [19,20]. Among them, DLD sorting technology has been widely used in the past decade in the fields of cell observation, cell analysis, and drug development due to its ease of operation, high sorting accuracy (resolution up to nanometer [21]), and the flexibility to adjust the size threshold when separating particles [20]. The DLD microfluidic chip for particle sorting consists of a flat microchannel and an array of pillars inside the channel at an angle to the flow direction. Particles smaller than the critical size do not move across the flow streamline and move in a zigzag mode, while particles larger than the critical size move with the array at different angles in a displacement mode. Huang et al. [22] first proposed DLD technology and successfully separated polymer particles with diameters of 0.9 μm and 1 μm at a flow velocity of 400 μm/s using this technique, achieving a sorting accuracy of 100% in experiments. In order to improve sorting efficiency and device throughput, many scholars have optimized the design of the pillars array’s shape, row shift fraction, tilt angle, and periodic arrangement. The shape of the pillar not only affects the streamline distribution in the flow field but also has a significant impact on the motion trajectories of particles in the DLD array (especially deformable particles), thereby directly influencing the separation efficiency of the chip. Therefore, many scholars, based on the research of traditional regular pillars [23] such as circular, triangular, and square shapes, have proposed irregular pillars such as L-shaped [24], asymmetric-shaped [25], airfoil-shaped [26], and I-shaped [27] and have conducted studies on the particle separation effects of different pillar shapes. Ranjan et al. [24] were the first to cut square pillars using different methods to create I-shaped, T-shaped, L-shaped, and Anvil-shaped pillars and conducted sorting experiments for rigid particles and erythrocytes. In the study, the authors introduced the separation index *I_s_* to quantify the sorting performance between different pillar designs. *I_s_* > 50 is considered a significant particle separation, and the higher the *I_s_*, the better the particle separation effect. Research shows that regardless of whether the particles are rigid or deformable, the separation index of the I-shaped pillar is the highest, exceeding 90, which is far superior to the circular pillar (with *I_s,rigid_* = 17.4 and *I_s,deformable_* = 0.3). Therefore, the I-shaped pillar is the most effective separation pillar shape among all the designs in this study. The research by Zeming et al. [27] further validates the advantages of the I-shaped pillar, indicating that this shape can fully utilize the maximum effective size of deformable particles. The groove structure induces particle flipping without deformation, thereby achieving stable and continuous flow separation of deformable particles towards the main channel side. In summary, the I-shaped pillars, due to their unique protrusions and groove structures, exhibit good separation efficiency in the sorting of rigid and deformable particles. Therefore, the study of I-shaped pillars is of great significance and deserves further in-depth exploration.

**Figure 1:**
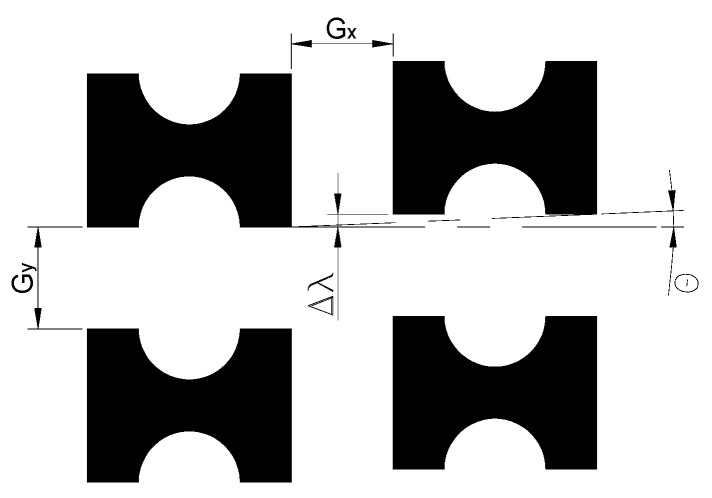
Design parameters of the pillar arrays. The lateral gap size (G_x_) and the downstream gap size (G_y_) can have different values. The offset angle of the array is θ, every row is shifted laterally Δλ compared to the previous row.

In microfluidic chips, the critical separation size is a key factor determining particle trajectories. The geometric design parameters of the chip (Figure 1) have a significant impact on the critical separation size. Although the empirical model of DLD has been widely studied and gradually improved, its research scope mainly focuses on spherical particles with regular geometrical shapes of pillar arrays. To predict particle separation in arrays with different pillar geometries, Zhang Z et al. [23] simulated circular, square, diamond, and triangular pillar arrays and derived a general equation for the critical separation size of different pillar shapes:

(1) $$D_{c}=\alpha G\varepsilon^{\beta }$$

This equation incorporates dimensionless geometric parameters, *α* and *β*, which vary with pillar geometry. Zeming et al. [28] experimentally demonstrated that optimizing the downstream gap size in circular pillar arrays by adjusting the lateral-to-downstream gap ratio can effectively enhance erythrocyte separation efficiency while maintaining high throughput. Rezaei et al. [29] obtained a new fitting formula for the critical separation size in asymmetric circular pillar arrays through numerical simulation. In addition, Vernekar et al. [30] conducted experimental studies on the motion trajectories of microbeads in different DLD arrays, focusing on analyzing the impact of anisotropy caused by changes in the shape and aspect ratio of the pillars on particle behavior. Although the studies have significantly expanded the applicability of the DLD empirical model, they mainly focus on circular pillar arrays with regular geometries. For irregular pillar arrays with complex structures (such as I-shaped pillars), the specific mechanisms by which the ratio of lateral gap to downstream gap (G_x_:G_y_) affects particle motion trajectories and critical separation sizes are still unclear. Additionally, since the DLD sorting size ranges from the submicron to nanoscale [20], the array channels are extremely narrow, and the flow remains in a low Reynolds number laminar state. As a result, throughput becomes one of the key design parameters for DLD chips. The variation in the G_x_ to G_y_ ratio directly affects the channel width, thereby influencing the device throughput.

In this paper, particle trajectory observation experiments and corresponding numerical simulations are carried out for I-shaped pillar arrays with G_x_:G_y_ >1. After the experimental results verified the accuracy of the numerical simulations, three chip arrays with different ratios of lateral gap to downstream gap (G_x_:G_y_) were further designed. By using a two-dimensional finite element method, we systematically analyzed the impact mechanism of the lateral-to-downstream gap ratio on device throughput and particle critical separation size. This study provides theoretical support for optimizing microfluidic chip design and improving particle separation efficiency.

## 2. Particle Trajectory Observation Experiment

### 2.1. Device Design and Fabrication

**Figure 2:**
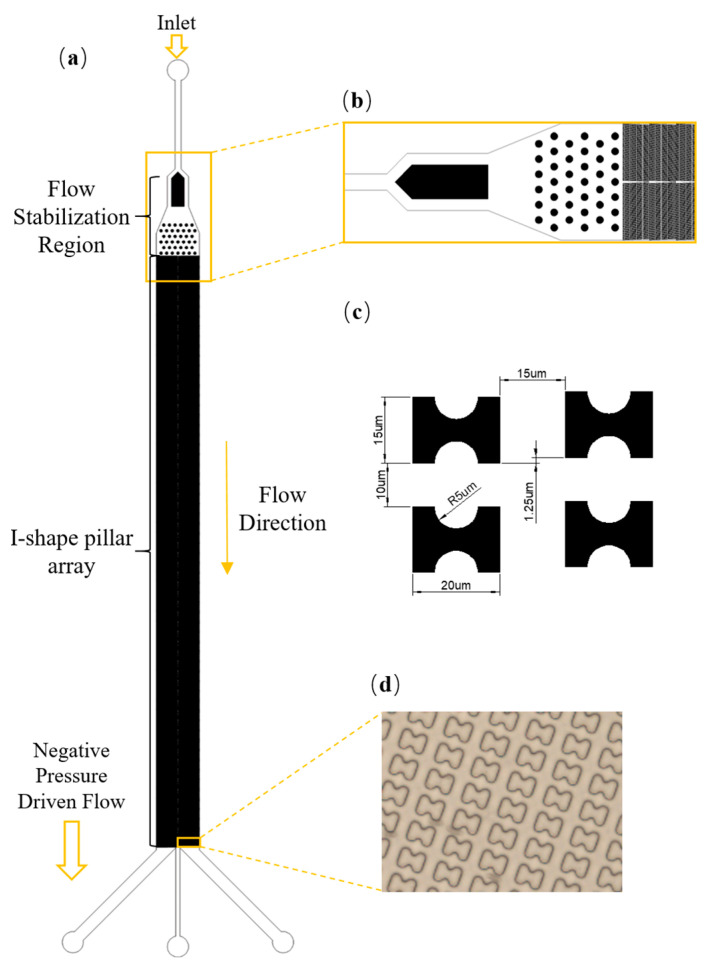
Device schematics. (**a**) The structural design of the device; (**b**) Schematic layout of rectifier array; (**c**) Geometric parameters of pillar arrays; (**d**) Schematic diagram of high-speed camera acquisition position.

The structure of the DLD chip designed for the experiment is illustrated in Figure 2. To enhance the uniform distribution of particles, the pressure-driven DLD microfluidic chip incorporates a rectifier pillar array between the inlet and the main pillar array. The designed DLD microfluidic chip consists of an inlet, a rectifier pillar array at the front section of the channel, a pillar array in the middle section, and three outlets at the end.

The DLD chip used in this paper has a length of L = 72.4 mm, a width of W = 24.9 mm, and an array configuration where the lateral gap between two pillars is G_x_ = 15 μm, the downstream gap G_y_ = 10 μm, and the row shift distance Δλ = 1.25 μm. The I-shaped pillars have a length of L_e_ = 20 μm, and a width of L_w_ = 15 μm. The semicircular grooves are centered at the midpoint of the pillar’s long edge, with a radius of R = 5 μm, and the channel height is H = 15 μm. To increase throughput, the pillars are arranged in a mirrored double-row structure, effectively doubling the array width without changing the channel length.

The microfluidic chip is fabricated using soft lithography: first, a template is fabricated using photoresist, then the template is replicated using PDMS material, and the top layer of the chip is formed, and finally the fabricated top layer is closed with a glass sheet to produce a complete chip.

### 2.2. Preparation of the Experiment

The particles used in this study were polystyrene microspheres with diameters of 1 μm, 3 μm, and 5 μm (Tianjin BaseLine ChromTech Research Centre, Tianjin, China). The particles were diluted with deionized water, and an appropriate amount of Tween-20 (Aladdin, Shanghai, China) was added. The mixture was then sonicated for 10 min to ensure good monodispersity. Finally, the size-specific solutions were individually sealed and stored for subsequent use.

Before the experiment, anhydrous ethanol was introduced into the channel for 10 min to perform a wetting pretreatment, ensuring the removal of air bubbles from the channel. To drive the flow process, the sample solution was first subjected to thorough sonication and homogenization. Then a 1000 μL gas-tight Hamilton glass syringe was used to aspirate the particle solutions with diameters of 1 μm, 3 μm, and 5 μm. The syringe was connected to a PTFE (polytetrafluoroethylene) tubing, which was then inserted into the outlet of the device. The syringe was connected to a syringe pump (HARVARD, Holliston, MA, USA), and the syringe piston was adjusted to inject the sample solution into the DLD chip at a constant flow rate of 20 μL/min. The flow rate was maintained consistently throughout the experiment to ensure stable pressure and avoid fluctuations. To capture the beads and red blood cells separated at the output of the DLD device, an Edmund Optics high-speed camera (PL-B742F) was connected to a DMI 3000b (Leica, Wetzlar, Germany) inverted bright-field microscope with a magnification of 200×. Particle trajectory images were acquired from the end of the array, and the collected particle images were processed using ImageJ (version 1.54f) software.

### 2.3. Experimental Results

**Figure 3:**
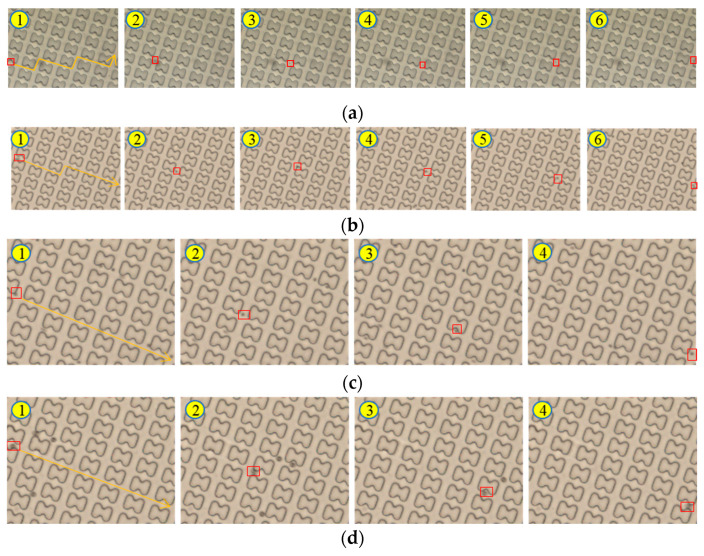
Plot of particle trajectories under 20 μL/min. (**a**) 1 μm particles in zigzag mode; (**b**) 3 μm particles in zigzag mode; (**c**) 3 μm particles in displacement mode; (**d**) 5 μm particles in displacement mode. (The red boxes indicate the position changes of the tracked particle at different time points).

To ensure the accuracy of the experimental results, three sets of experiments were conducted, and post-processing of the experimental data was performed, as shown in Figure 3. Under the condition of a 20 μL/min flow rate, the trajectories of particles with different sizes in the chip showed significant differences.

1 μm particles adhered closely to the pillars, moving along the first streamline, and exhibiting stable zigzag trajectories. Due to the rectification array at the chip inlet, the 1 μm particles were evenly distributed within the chip, and particle images could be collected at the outlets of all channels.

In contrast, 5 μm particles maintained a displacement mode of movement after colliding with the pillars. As the chip was designed with a symmetric mirror structure, the trajectories of the 5 μm particles gradually converged toward the chip center, forming a highly concentrated distribution at the outlet. Particle images could only be collected from the central channel, exhibiting a significant focusing effect.

3 μm particles displayed a more complex motion pattern, exhibiting two distinct trajectory forms. Most of the time, they crossed streamlines in a displacement mode (Figure 3c); however, in rare cases, they switched to a zigzag mode (Figure 3b). At the outlet, 3 μm particles were mainly concentrated in the central channel, but their focusing effect was significantly weaker than that of 5 μm particles, with a relatively broader aggregation region.

The experimental results indicate that the critical separation size of the chip is approximately 3 μm. Based on this analysis, a more detailed investigation of the chip’s critical separation size will be conducted to further optimize chip performance and particle separation efficiency.

## 3. Simulation

First, we simulate and validate the flow in the experiment using the finite element method. For the simulations, we have used COMSOL Multiphysics 6.3 to model the particle movement in DLD arrays. The two-dimensional (2D) Finite Element Method (FEM) was used by adding the Peristaltic Flow module and the Solid Mechanics module, and to improve the accuracy of the particle trajectories, a bi-directional FSI model was constructed of the particles moving within the I-shaped pillar array. The peristaltic flow module and solid mechanics module were employed, with a two-dimensional (2D) FEM approach. To enhance the accuracy of particle trajectory predictions, a bidirectional fluid-structure interaction (FSI) model was constructed to capture the motion of particles within the I-shaped pillar array. Periodic conditions are added to the top and bottom boundaries of the model to maintain equal velocity and pressure in the flow field. The two-dimensional particle model is constructed, and the shallow channel approximation theory is introduced to introduce the effect of channel height on the flow into the equations in the form of volumetric force.

**Figure 4:**
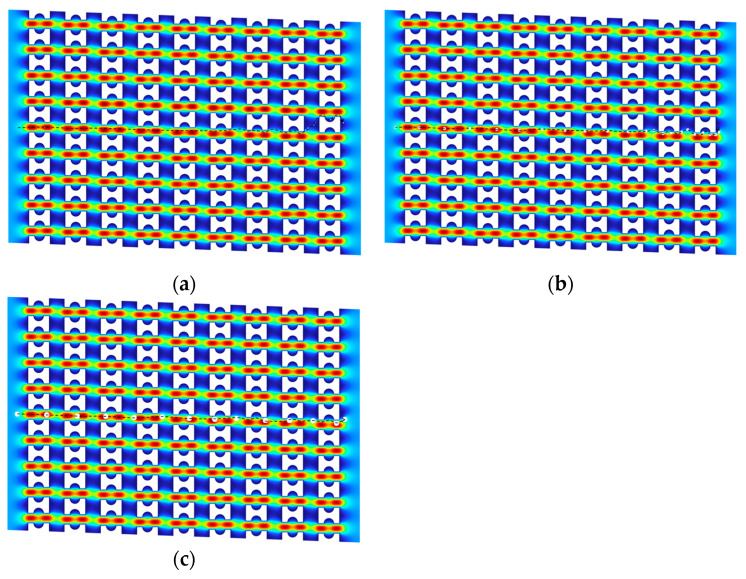
Diagram of the trajectory of a particle in one cycle: (**a**) 1 μm particle; (**b**) 3 μm particle; (**c**) 5 μm particle. (The dashed lines represent the trajectory of the particle in the flow field).

**Figure 5:**
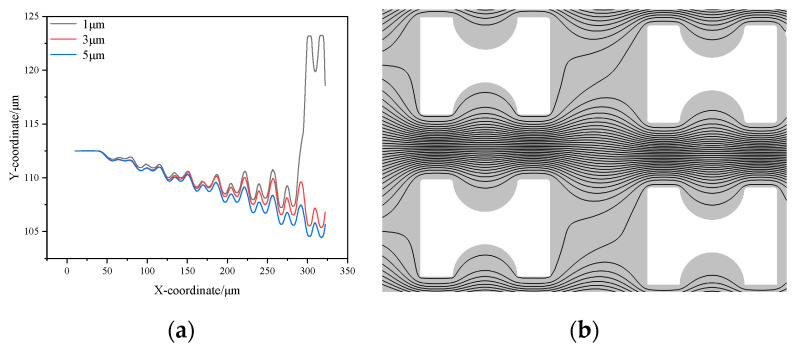
Diagram of particle coordinate and streamline in the array: (**a**) particle coordinate; (**b**) streamline.

In our simulation, we first constructed and modeled the motion behavior of 1 μm, 3 μm, and 5 μm particles in an array of G_x_ = 15 μm versus G_y_ = 10 μm (G_x_:G_y_ > 1) pillars used in the experiment shown for one flow cycle to verify the reliability of the simulation. In this setup, the I-shaped pillars were assumed to be rigid bodies, and for the particle characteristics, we used polystyrene mechanical properties. The fluid properties of water at room temperature were used to model the fluid. To study the interaction mechanisms more comprehensively between the fluid and particles, three types of particles were simulated, each entering the array from the central region of the pillar array outside the flow field. As shown in Figure 4, after entering the array, the three types of particles gradually shifted upward under the influence of the fluid, eventually approaching the surface of the pillars in the previous row. When the fluid passes through the lateral gap between adjacent pillars, it splits into two branches. As observed from the streamline diagram (Figure 5b), the fluid near the pillars is deflected upward due to the influence of the flow field on the pillar surface, passing over the top of the next pillar—this flow was defined as “upward flow”. In contrast, the fluid farther from the pillar surface passed directly through the gap below the pillar, defined as “direct flow”. The combined effects of these two flow patterns created a complex flow field environment, resulting in differentiated motion behaviors for particles of different sizes. The 1 μm particles exhibited a clear zigzag mode within the periodic pillar array, with their overall movement path consistently confined to the pillar array in the current row, without crossing into adjacent rows. This stable motion pattern was primarily attributed to the small size of the 1 μm particles. As the particles gradually approached the pillars, they remained under the significant influence of the first streamline. Due to the strong upward flow effects exerted by the fluid, the 1 μm particles were unable to escape the influence of this flow stream and continued to move along an upward trajectory.

The 3 μm particles exhibited a displacement mode, crossing flow streams at the end of the array and transitioning from the current row to the next row of pillars. Observing their trajectories revealed a gradual increase in upward displacement tendency within a single cycle. This indicates a certain degree of instability in the motion pattern of the 3 μm particles, particularly when approaching the pillars, where they were significantly disturbed by the first streamline. The 5 μm particles also displayed a displacement mode; however, their trajectories were more stable compared to the 3 μm particles. Starting from the fifth column of pillars in the array, the trajectories of the 5 μm particles became increasingly stable (Figure 5a), maintaining this consistency through to the end of the array. In conclusion, the simulation results were highly consistent with the experimental observations. The study demonstrates that the pillar array design effectively separates 1 μm, 3 μm, and 5 μm particles, with a critical separation diameter of approximately 3 μm.

**Figure 6:**
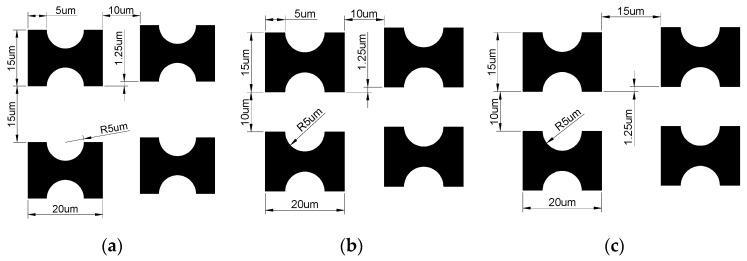
Three different array configurations: (**a**) G_x_:G_y_ > 1; (**b**) G_x_:G_y_ = 1; (**c**) G_x_:G_y_ < 1.

Based on this design, pillar arrays with G_x_:G_y_ = 1 and G_x_:G_y_ < 1 were constructed (as shown in Figure 6), and the impact of different arrangement patterns on device throughput and critical separation size was further investigated. Under the condition of maintaining a constant flow rate in each channel, steady-state flow fields for three different arrangement patterns were simulated, and the effect of the G_x_ to G_y_ ratio on device throughput was analyzed through the pressure difference within a single period.

The simulation study for the critical separation size of the array is performed on a specific localized region of the whole flow field. In order to avoid the influence of sidewall effects on the particle trajectories and to allow direct observation of the results of the particle interaction with the first flow beam, the particles start their motion close to the pillars but not immediately on the walls.

Based on the study, the critical separation size of the G_x_:G_y_ > 1 array is around 3 μm. Therefore, five different sizes of particles, 1.5 μm, 2 μm, 2.5 μm, 3 μm, and 3.5 μm, were selected for the simulation, and the inlet flow rate and period were set up according to the array arrangement as shown in Table 1.

The particle motion pattern is determined by the path of the particles in the second pillar. If the particles follow the first flow streamline and pass above the second pillar, it is a zigzag mode. If the particles deviate from the first flow streamline and continue moving below the second pillar, it is a displacement mode.

## 4. Results

### 4.1. Device Throughput

The throughput of a DLD device is influenced by the fluid resistance of the entire array—the lower the resistance, the higher the throughput. In microfluidic systems, fluid resistance is typically calculated as the ratio of pressure difference (Δ*P*) to flow rate (*Q*), expressed mathematically as:

(2) $$R=\frac{\Delta P}{Q}$$

**Figure 7:**
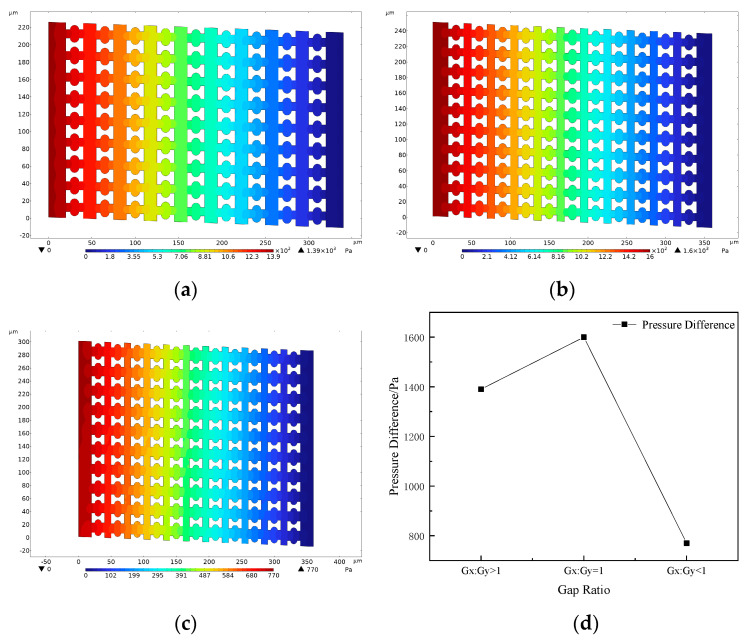
Diagram of Pressure distribution in the array: (**a**) G_x_:G_y_ > 1; (**b**) G_x_:G_y_ = 1; (**c**) G_x_:G_y_ < 1; (**d**) Differential pressure in three arrays.

Since the flow rate into each pillar gap is equal, the flow resistance of the device can be evaluated by the magnitude of the pressure difference Δ*P*. Figure 7 shows the pressure distribution of the three arrays over one cycle. The steady-state simulation results indicate that the fluid pressure inside the pillar array gradually decreases in the flow direction, presenting an overall steady-state pressure difference trend.

A comparison of the pressure differences of the three arrays shows that the array arrangement with G_x_:G_y_ < 1 has the lowest pressure difference of 770 Pa, the array with G_x_:G_y_ = 1 has the highest resistance of 1600 Pa, and the array with G_x_:G_y_ > 1 has a slightly lower pressure difference of 1390 Pa than that of G_x_:G_y_ = 1. In addition, the pressure difference increases by 830 Pa when G_x_ is kept constant and G_y_ decreases by 5 μm, while it increases by only 210 Pa when G_y_ is held constant and G_x_ is decreased by 5 μm; the pressure difference increases by only 210 Pa. This suggests that the array throughput is jointly affected by G_x_ size and G_y_ size but is mainly controlled by G_y_ size. This result is consistent with the simulation study by Zeming et al. [28], who analyzed flow resistance through flow field velocity distribution. They constructed three different circular pillar arrays with G_x_:G_y_ ratios of 15:2, 15:15, and 2:15, obtaining flow field velocity distribution maps for each array and analyzing the distribution of high-velocity and low-velocity regions. The study demonstrated that optimizing the downstream and lateral gap sizes effectively reduces flow resistance, thereby enhancing device throughput and sorting performance.

This phenomenon is mainly due to the fact that most of the fluid entering the pillar array flows along the main channel and is discharged in the direction of the array offset, while G_y_ directly determines the width of the main channel. The wider the main channel, the lower the flow resistance, and the corresponding increase in throughput. At the same time, G_x_ also has a certain impact on the flow rate: when G_x_ increases, the upward flow channel widens, reducing the overall flow resistance and thereby increasing the flow rate. Therefore, when optimizing the DLD array design, it is necessary to comprehensively consider the ratio of G_x_ to G_y_ in order to achieve a reasonable balance between separation accuracy and throughput.

### 4.2. Critical Separation Size

**Figure 8:**
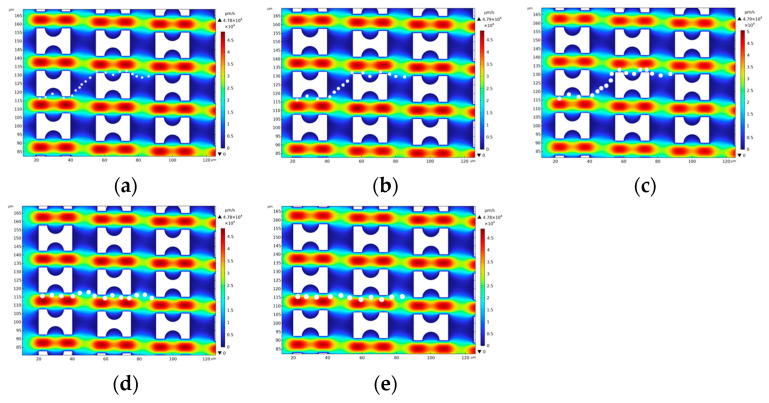
G_x_:G_y_ > 1 Array Particle Trajectory Diagram: (**a**) 1.5 μm; (**b**) 2 μm; (**c**) 2.5 μm; (**d**) 3 μm; (**e**) 3.5 μm.

**Figure 9:**
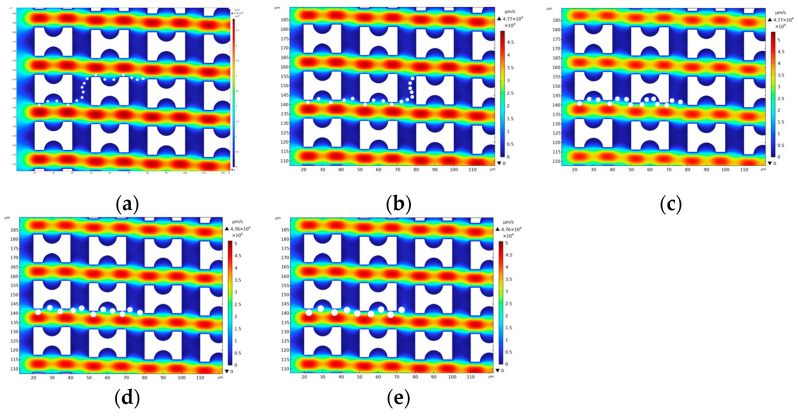
G_x_:G_y_ = 1 Array Particle Trajectory Diagram: (**a**) 1.5 μm; (**b**) 2 μm; (**c**) 2.5 μm; (**d**) 3 μm; (**e**) 3.5 μm.

**Figure 10:**
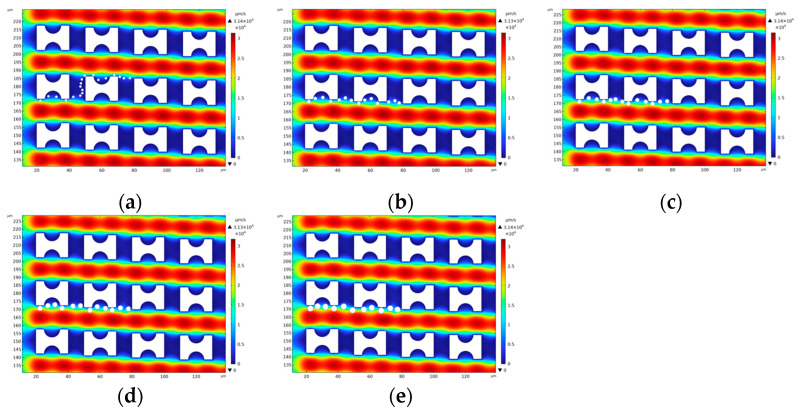
G_x_:G_y_ < 1 Array Particle Trajectory Diagram: (**a**) 1.5 μm; (**b**) 2 μm; (**c**) 2.5 μm; (**d**) 3 μm; (**e**) 3.5 μm.

The distribution of particle motion patterns is significantly affected by the geometry of the flow field. Figure 8, Figure 9 and Figure 10 show the motion trajectories of particles in the flow field for three arrays, G_x_:G_y_ > 1, G_x_:G_y_ = 1, and G_x_:G_y_ < 1, respectively. In these arrays, the ratio of the lateral gap to the downstream gap directly determines the motion patterns of the particles and their critical sizes. The motion patterns of five particles in the three types of arrays are organized as shown in Table 2.

**Figure 11:**
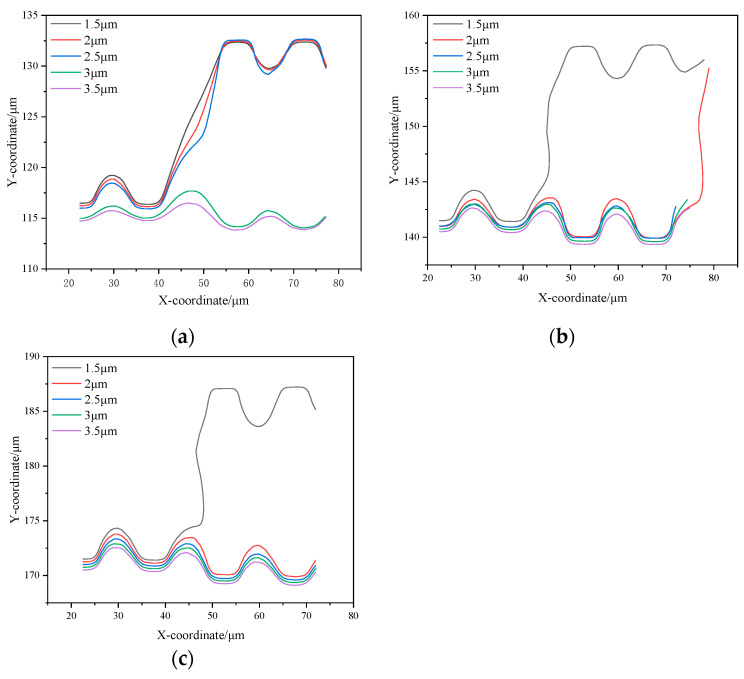
Diagram of particle coordinates in the array: (**a**) G_x_:G_y_ > 1; (**b**) G_x_:G_y_ = 1; (**c**) G_x_:G_y_ < 1.

In the G_x_:G_y_ > 1 array, the 1.5 μm, 2 μm, and 2.5 μm diameter particles are in a zigzag mode. Due to the initial position of the particles close to the wall, they are significantly affected by the first flow streamline at the beginning of the flow, and it can be observed that their trajectories fully enter the semicircular notch region of the first pillar. Subsequently, these particles are shifted upward by the upward flow influence as they flow through the transverse gap between the first and second pillars and exit the flow field from the top of the second pillar. In contrast, the 3 μm and 3.5 μm diameter particles, due to their larger sizes, were able to escape from the restriction of the upward-shifted flow and flowed out of the flow field from the lower part of the second pillar, which showed a displacement mode. Figure 11a–c show that the larger the particle diameter, the easier it is to escape from the control of the first flow streamline; e.g., a 1.5 μm particle will completely enter the semicircular groove of the first pillar, whereas a 3.5 μm particle exhibits only a small upward shift before being driven by the fluid to continue to move in the lateral direction.

In the G_x_:G_y_ = 1 array, the lateral gap is significantly reduced compared to the G_x_:G_y_ > 1 array. This change in geometry results in a change in the pattern of the particles subjected to the fluid. Specifically, the particles with diameters of 1.5 μm and 2 μm maintain a zigzag mode, but the particles with diameters of 2.5 μm, 3 μm, and 3.5 μm are in a displacement mode. The simulation results are consistent with the study by Zeming et al. [27] in Table 3. They conducted a sorting experiment on polystyrene (PS) particles using an I-shaped pillar array with a characteristic length of 15 μm and G_x_ = G_y_ = 10 μm. In the experiment, particles with diameters ranging from 2.0 to 3.5 μm in steps of 0.5 μm were introduced into the DLD chip. It was observed that 2.5 μm particles exhibited noticeable trajectory shifts, while 3.0 μm particles achieved complete separation. The experimental results indicate that the critical separation diameter of this array is approximately 2.5 μm, which aligns closely with the simulation results of this study, further validating the accuracy of our findings. The narrowing of the lateral gap weakens the upward shifting effect of the fluid, limiting the ability of the particles to deflect upward, thus further reducing the critical size compared to the G_x_:G_y_ > 1 array.

In the G_x_:G_y_ = 1 array, the lateral gap is significantly reduced compared to the G_x_:G_y_ > 1 array. This change in geometry results in a change in the pattern of the particles subjected to the fluid. Specifically, the particles with diameters of 1.5 μm and 2 μm maintain a zigzag mode, but the particles with diameters of 2.5 μm, 3 μm, and 3.5 μm are in a displacement mode. The narrowing of the lateral gap weakens the upward shifting effect of the fluid, limiting the ability of the particles to deflect upward, thus further reducing the critical size compared to the G_x_:G_y_ > 1 array.

The critical size of the G_x_:G_y_ < 1 array is further reduced, with only the 1.5 μm diameter particles showing a zigzag mode, while larger particles moved in a displacement mode, displaying similar trajectories (Figure 11c).

In summary, the motion pattern of the particles is determined by both their size and the geometry of the flow field. Smaller particles are more likely to be controlled by the first flow streamline and show zigzag mode, while larger particles tend to be free from the influence of the upward-moving flow and show displacement mode. The geometrical parameters of the array (especially the ratio of the lateral gap to the downstream gap, G_x_:G_y_) have a strong influence on the particle motion pattern and critical size. As G_x_:G_y_ decreases, the ability of the particles to be subjected to fluid upward flow decreases, and the critical size decreases accordingly. Behnam et al. [29] adjusted the G_x_:G_y_ ratio by keeping the lateral gap G_x_ of circular pillars constant while increasing the downstream gap G_y_ to investigate the effect of array asymmetry on the critical separation diameter. Consistent with the findings of this study, their results demonstrated that reducing the G_x_:G_y_ ratio enhances the separation capability of the array and improves sorting efficiency. Building on this, the present study further proposes the underlying mechanism of this phenomenon, revealing the impact of the G_x_-to-G_y_ ratio on the flow field distribution. It also explains how adjusting the main channel width and upward flow path can optimize the device throughput. The G_x_:G_y_ > 1 array has the most significant upward flow effect and is suitable for separating particles of smaller sizes, whereas the G_x_:G_y_ < 1 array has the lowest critical size and is more suitable for separating particles of larger sizes. The critical separation sizes of the three chips are close but different, suggesting some selectivity in their ability to separate particles or cells. This feature allows the user to choose the most suitable chip design for specific cell separation needs to optimize separation efficiency and precision.

### 4.3. Particle Trajectory

The previous analysis examined the influence of the G_x_:G_y_ ratio on the critical separation diameter and particle motion behavior. However, the underlying mechanisms remain inadequately explained. To further investigate its effects, this section provides a detailed analysis from two perspectives: streamline distribution and velocity field characteristics, aiming to uncover the intrinsic mechanisms by which changes in the G_x_:G_y_ ratio impact particle motion patterns.

**Figure 12:**
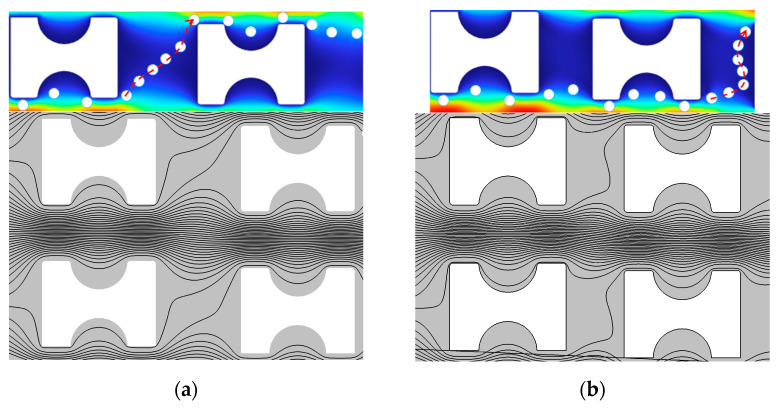

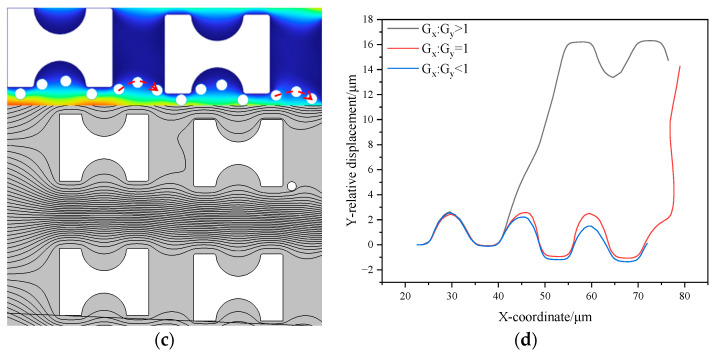
2 μm particle motion trajectory diagrams, flow line diagrams, and relative displacement diagrams within three arrays: (**a**) G_x_:G_y_ > 1; (**b**) G_x_:G_y_ = 1; (**c**) G_x_:G_y_ < 1; (**d**) 2 μm particle relative displacement diagram within three arrays.

The trajectories of particles with a diameter of 2 μm in the three types of chips show significant differences. Therefore, in this study, particles with a diameter of 2 μm are selected as the object of analysis and are explored by deriving streamline diagrams in the array. In order to ensure that the sparsity of the streamlines is consistent, the same sampling frequency is used for all streamline diagrams. By comparing the particle trajectories (Figure 12 labeled by the red dashed line in the middle) with the streamline diagrams, we can see:

In the G_x_:G_y_ > 1 and G_x_:G_y_ = 1 arrays, the 2 μm particles exhibit a zigzag mode between the first and second pillars, with their trajectories closely matching the streamlines of the upward flow. This phenomenon aligns with the fundamental theory of DLD separation, indicating that the particles in this region are primarily influenced by the upward flow.

In contrast, in the G_x_:G_y_ < 1 array, although the 2 μm particles are influenced by the upward flow and show a certain upward motion trend in the pillar gaps, the insufficient flow rate of the upward flow prevents the particles from reaching the upper edge of the second pillar. Consequently, the particles exhibit displacement mode instead. This indicates that the flow rate of the upward flow plays a critical role in determining the transition of particle motion modes.

The simulation allows us to derive the proportion of upward flow to the total flow for the three arrays. Total flow is the sum of the upward flow and the direct flow, as shown in Table 4. Combined with the analysis of particle velocity diagrams, flux distribution, and streamline diagrams, the mechanism of the G_x_:G_y_ ratio on the flux of the upward stream and the particle motion behavior can be further revealed.

In the G_x_:G_y_ > 1 array, the flux shares of the upward flow reach 6.70% of the total flow, which is the largest among the three arrays. As the G_x_:G_y_ ratio decreases, the flux of the upward stream gradually decreases. In all three arrays, although the total flow rate at the inlet is constant and the same, the flow share of the upward flow changes significantly due to the different geometrical parameters of the lateral gap (G_x_) to downstream gap (G_y_), which affects the particle motion pattern.

**Figure 13:**
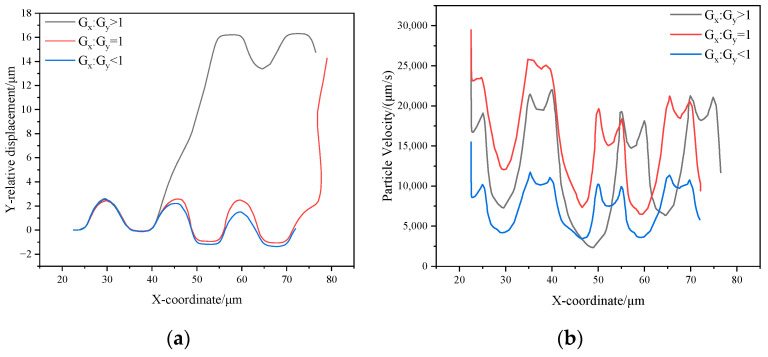
Diagram of relative displacement and particle velocity within three arrays: (**a**) Relative displacement; (**b**) Particle velocity.

In the G_x_:G_y_ = 1 array, the downstream gap (G_y_) remains the same (15 µm) as G_x_:G_y_ > 1, but the lateral gap (G_x_) decreases from 15 µm to 10 µm. This change leads to a narrowing of the flow channel for the upward-moving flow, and consequently, a decrease in the upward flux share; at the same time, an increase in the flux of the direct flow leads to a significant acceleration of the particles’ velocity, as shown in Figure 13b, which shows that the velocity of particles at the gap between the first and second pillars is significantly higher compared to the G_x_:G_y_ > 1 array. The increase in particle velocity makes it easier for them to escape from the first flow streamline and thus change their motion pattern. As the particle passes through the lateral gap of the pillar and interacts with the pillar, its velocity gradually decreases to a level like that of the G_x_:G_y_ > 1 array. In this case, the particle can re-expose itself to the upward flow after leaving the second pillar and complete the upward motion. This mechanism effectively explains the behavior of the 2 µm particles in the G_x_:G_y_ = 1 array, which can follow the upward flow and complete the upward movement only after reaching the second pillar.

Compared with the G_x_:G_y_ = 1 array, the lateral gap (G_x_) of the G_x_:G_y_ < 1 array remains unchanged (10 µm), but the downstream gap (G_y_) is increased from 10 µm to 15 µm. The particle velocity is drastically reduced due to the increase in the flow channel of the direct flow; at the same time, the flux of the upward flow is further reduced, and the ratio of the upward flow to the total flux is only 2.19%. As shown in Figure 12a–c and Figure 13b, even though the particle velocity is greatly reduced, the influence of the upward flow in the flow field almost completely disappears, leading to a significant weakening of the upward motion tendency of the particles, which ultimately leads to a further reduction in the critical separation size.

The G_x_:G_y_ ratio has a decisive influence on the upward flow ratio and the flow velocity in the flow field, while the flow velocity and the upward flow directly determine the particle motion pattern and the change of the critical separation size. The upward flow of particles is strongest in the G_x_:G_y_ > 1 array, which is suitable for separating larger particles, while the upward flow almost disappears in the G_x_:G_y_ < 1 array, which is suitable for the separation of smaller particles. These results provide an important theoretical basis for optimizing the chip design to meet different separation needs.

## 5. Conclusions

DLD technology is a passive microfluidic sorting method based on particle size differences, where particles in the array mainly exhibit two types of movement modes: particles smaller than the critical separation diameter move along a zigzag mode, while particles larger than the critical separation diameter shift in displacement mode. The critical separation diameter and throughput are key parameters that determine the sorting performance of DLD chips and are influenced by factors such as pillar shape and arrangement. Among these, the asymmetry between the array’s lateral gap (G_x_) and downstream gap (G_y_) leads to changes in the critical separation diameter, thereby affecting the chip’s sorting efficiency.

This study combines experiments and numerical simulations to systematically explore the effects of different lateral-to-downstream gap ratios (G_x_:G_y_) on device throughput, particle movement modes, and critical separation diameter. The throughput of the DLD device is characterized by measuring the pressure difference over one period under the same inlet flow conditions, where a lower pressure difference indicates higher throughput. The results show that the throughput of the DLD device is influenced by both G_x_ and G_y_, with G_y_ being the dominant factor. Increasing G_y_ significantly enhances the throughput. This conclusion is consistent with the findings of Zeming et al. [28], who reached similar results based on flow field velocity distribution analysis. Although the critical separation sizes of the three chips are close to each other, there are still some differences, and such differences provide the possibility for users to choose the most suitable chip design according to the specific cell separation needs, thus achieving the optimization of separation efficiency and precision.

Meanwhile, the decisive influence of the G_x_:G_y_ ratio on the percentage of upward-moving flow within the flow field and the velocity of the flow field is further revealed through the comprehensive analysis of the flow distribution, streamline characteristics, and particle velocity field of the pillar array. Specifically, the decrease in the G_x_:G_y_ ratio directly leads to the decrease in the upward flux and the change of the flow velocity, which in turn affects the particle motion pattern and the critical separation size. The lower the G_x_:G_y_, the more the particles tend to move in the displacement mode rather than the zigzag mode. This mechanism suggests that the G_x_:G_y_ ratio realizes the precise control of particle motion behavior by modulating the hydrodynamic properties, which lays the foundation for further promoting the application of microfluidic technology in biomedical fields.

## Figures and Tables

**Table 1:** Volume Rate of Flow, array offset angle, and cycle for three chips.

Microchip	Volume Rate of Flow	Gradient (θ)	Cycle
G_x_:G_y_ > 1	2.629 μL/min	2	9
G_x_:G_y_ = 1	2.9211 μL/min	2	11
G_x_:G_y_ < 1	2.9211 μL/min	2	11

**Table 2:** Motion patterns of the five particles.

Microchip	1.5 μm	2 μm	2.5 μm	3 μm	3.5 μm
G_x_:G_y_ > 1	zigzag	zigzag	zigzag	displacement	displacement
G_x_:G_y_ = 1	zigzag	zigzag	displacement	displacement	displacement
G_x_:G_y_ < 1	zigzag	displacement	displacement	displacement	displacement

**Table 3:** Three types of array critical separation diameters and Zeming experimental values.

Microchip (G_x_:G_y_)	15:10	10:10	10:15	Zeming’ Experiment
critical separation diameter	2.5–3 μm	2–2.5 μm	1.5–2 μm	2 μm

**Table 4:** Proportion of upward flow in the three arrays.

Microchip	Total Flow	Upward Flow	Proportion
G_x_:G_y_ > 1	0.29342 μL/min	0.02045 μL/min	6.7%
G_x_:G_y_ = 1	0.29284 μL/min	0.01530 μL/min	5.22%
G_x_:G_y_ < 1	0.29354 μL/min	0.00643 μL/min	2.19%

## Data Availability

The data that support the findings of this study are available on request from the corresponding author.
